# Three-dimensional speckle tracking echocardiography for evaluation of ventricular function in patients with systemic lupus erythematosus: relationship between duration of lupus erythematosus and left ventricular dysfunction by using global longitudinal strain

**DOI:** 10.1186/s43044-024-00511-4

**Published:** 2024-06-24

**Authors:** Nehzat Akiash, Somayeh Abbaspour, Karim Mowla, Amir Moradi, Shahla Madjidi, Parisa Sharifi, Mahboubeh Pazoki

**Affiliations:** 1 Atherosclerosis Research Center, Ahvaz Jundishapur University of Medical Sciences, Golestan Blvd., Ahvaz, Iran; 2 Department of Rheumatology, Golestan Hospital, Ahvaz Jundishapur University of Medical Sciences, Ahvaz, Iran; 3 Department of Cardiology, Nikan Hospital, Tehran, Iran; 4 Department of Cardiology, School of Medicine, Hazarat-e Rasool General Hospital, Iran University of Medical Sciences, Tehran, Iran

**Keywords:** Systemic lupus erythematous, Speckle tracking echocardiography, Left ventricular function, Global longitudinal strain, 3D echocardiography

## Abstract

**Background:**

Cardiovascular diseases are leading causes of morbidity and mortality in patients with systemic lupus erythematosus (SLE). Cardiac involvement in SLE can often go undetected. Three-dimensional (3D) speckle tracking echocardiography (STE) is a noninvasive imaging technique that can assess the function of the heart’s ventricles in an accurate and reproducible way. This makes it an attractive option for detecting early signs of heart disease in SLE patients. By identifying these subclinical cardiac abnormalities, 3D-STE may help reduce the negative impact of cardiovascular diseases in SLE population. Therefore, this study aimed to compare the left ventricular (LV) function between patients with SLE compared to age- and gender-matched controls using two-dimensional (2D) and 3D-STE.

**Results:**

The current study found no significant differences in left ventricle ejection fraction, left ventricle end-diastolic volume, left ventricle end-systolic volume, left ventricle end-diastolic mass, and left ventricle end-systolic mass between the two groups. However, the SLE group exhibited a significantly lower LV global longitudinal strain (GLS) compared to the control group according to all types of echocardiographic assessments, including 3D and 2D long-axis strain, apical 2-chamber, and apical 4-chamber assessments (all *P* values < 0.05). Furthermore, a good inter-rater reliability and intra-rater reliability were observed regarding the LVGLS measurement with 3D-STE. Additionally, the study identified a significant correlation between LVGLS and SLE duration (*r* (50) = 0.46, *P* < 0.001). The use of prednisolone and nephrology disorders was also found to impact LVGLS measurements.

**Conclusions:**

Despite a normal LVEF in patients with SLE, LVGLS measurements indicated that LV systolic dysfunction was observed more frequently in SLE patients compared to their healthy counterparts. Therefore, advanced 3D-STE techniques may be useful in identifying subtle abnormalities in LV function in SLE patients.

## Background

Systemic lupus erythematosus (SLE) is a chronic, relapsing, inflammatory connective tissue disorder resulting in multi-organ involvement, including the skin, kidney, and serosal membranes [1]. Although SLE can affect individuals of any age or gender, it is more commonly observed in young women [2]. Despite extensive research, the precise etiology of SLE remains unclear, with genetic, environmental, and infectious factors all playing a potential role [3]. It is well established that SLE associates with cerebrovascular accidents [4, 5] and cardiovascular diseases (CVDs) [6, 7], increasing the risk of myocardial infarction (MI) by 10 times compared to the general population [8] and making CVDs one of the leading causes of death among these patients [9]. Contributing factors to this increased risk include immune dysregulation, endothelial dysfunction, defective vascular repair mechanisms [10], as well as classic risk factors of CVDs [8].

SLE patients frequently present with a variety of cardiac manifestations, including coronary artery disease (25–45%), Libman–Sacks endocarditis (13–74%), pericarditis (12–24%), myocarditis (10–40%), congestive heart failure (7–36%), and cardiac tamponade (< 3%) [11]. Recent meta-analyses have also shown a high prevalence of left ventricle (LV) dysfunction among SLE patients, even with a normal left ventricular ejection fraction (LVEF) [12–14]. Despite advances in medical treatments for SLE, their CVD-related mortality has remained unchanged, leading to considerable challenges in predicting and managing cardiac issues [15]. Moreover, traditional approaches to risk assessment, such as the Framingham risk score, have limited utility in predicting CVD events among SLE patients [8]. Consequently, there is an imperative need for refined techniques to evaluate cardiac function with greater precision in this population. Cardiac magnetic resonance (CMR) [16, 17] and tissue Doppler imaging (TDI) [18] have been proposed as viable methods for detecting subclinical CVDs in SLE patients. However, CMR is not generally employed due to its time-consuming nature and high costs [14]. On the other hand, while TDI is more acceptable, its results might be less reproducible for basal segments of the heart since it is angle-dependent and vulnerable to the force of surrounding tissues [19]. Two-dimensional (2D) speckle tracking echocardiography (STE) is an alternative noninvasive method, but it is prone to out-of-plane motion, limiting its reproducibility [20]. In contrast, three-dimensional (3D) STE has emerged as a promising, noninvasive, cost-effective, and precise technique for evaluating cardiac function. Unlike 2D-STE, the 3D-STE approach enables the tracking of speckle patterns that move out of the echocardiographic imaging plane, resulting in improved reproducibility and accuracy [21].

In light of this information, the current study aimed to evaluate the LV function of SLE patients using the novel and reproducible 3D-STE technique to improve the early detection and management of CVD in this population.

## Methods

The current case–control study was performed between September 2016 and March 2017. Patients with SLE who met the inclusion criteria were recruited and were compared with a control group of healthy individuals.

### Inclusion criteria

- SLE diagnosis was made at least three years ago
- Aged more than 18
- No history of prior known cardiovascular diseases
- Being interested in participating in the study

### Exclusion criteria

- Any abnormality in electrocardiogram or chest X-ray
- The existence of any cardiac murmur or extra sounds in cardiac auscultation
- Patients with an improper full-volume view of 3D-STE

Following a thorough physical examination and a review of medical records by a rheumatologist, the SLE group participants were selected according to the updated American College of Rheumatology criteria for SLE diagnosis [22, 23]. A total of 106 participants, consisting of 53 SLE patients and 53 healthy controls, were included in this study. One participant in the SLE group was excluded due to inadequate echocardiographic views. The SLE and control groups were matched for age and gender.

### Data collection

All patients underwent a comprehensive evaluation for rheumatologic and cardiovascular status. The SLE group’s medical history, drug history, and systemic involvement data were obtained from their medical records. Data on SLE risk factors, such as age, gender, history of hypertension, hyperlipidemia, smoking, and family history of SLE, as well as current SLE-related medications, symptoms, disease duration, and laboratory tests, were collected through face-to-face interviews and review of patients’ medical records.

### Echocardiographic assessments

Participants underwent comprehensive echocardiography using 2D-STE, 3D-STE, and TDI imaging with speckle tracking analysis to assess LV parameters, such as LVEF and LV size. A Vivid E9 ultrasound machine (GE Vingmed Ultrasound, Horten, Norway) equipped with a 3.5-MHz 4V-D cardiac sector probe was utilized for transthoracic echocardiography. In accordance with the guidelines set forth by the American Society of Echocardiography, volumetric echocardiographic data were collected over 4 to 6 cardiac cycles using a zoomed apical 4-chamber view of the LV (A4C) [24]. Subsequently, 4D Auto LVQ software (EchoPAC BT13, GE Vingmed Ultrasound, Horten, Norway) was utilized for volume analysis to determine left ventricular end-diastolic volume (LVEDV), left ventricular end-systolic volume (LVESV), and LVEF. Three points for each apical plane were required, consisting of two points at the edges of the mitral annulus and one at the apex, initially in end-diastolic frames and subsequently in end-systolic frames. The software automatically delineated the LV endocardial border in a 3D model from the end-diastolic and end-systolic phases, and manual adjustments were made when required due to inadequate automatic delineation. Left ventricular global longitudinal strain (LVGLS) was measured by performing a second epicardial tracking, and LV mass and strain were assessed by automatically delineating the region of interest. The software automatically determined the LVGLS and borders, and manual adjustments were made if the automatic delineation was deemed inaccurate.

An automated functional imaging method was used for 2D-STE LVGLS measurements. Three separate apical views, including the A4C, apical 2-chamber (A2C), and apical long-axis (LAX) views, were recorded for every patient, with a minimum frame rate of 50 frames per second. The margins of the endocardium were automatically demarcated in each picture, and the mitral annulus and LV apex were located. The region of interest was manually adjusted by the operator if needed. The mobility of the myocardium within each area of focus was then evaluated using STE. Each ventricular segment’s peak systolic longitudinal strain was calculated, and the results were combined into a bull’s-eye template using a 17-segment model. By computing the mean longitudinal strain across each of the 17 segments, the average global longitudinal peak systolic strain for the complete LV was calculated [25].

Moreover, to evaluate inter- and intra-rater variability as an indicator of method reproducibility, 26 randomly selected patients from the SLE group underwent 3D-STE the following week by both the first assessor and another cardiologist. The second cardiologist was blind to the previous echocardiographic measurements of patients.

### Statistical analysis

Descriptive statistics are used to present the data, with frequencies and percentages for categorical variables and means and standard deviations for continuous variables. The normality of the data distribution and the equality of variances were assessed using the Kolmogorov–Smirnov and Levene’s tests, respectively. The inter-rater reliability and intra-rater reliability of the measurements were evaluated using the intraclass correlation coefficient (ICC) with a two-way fixed model of absolute agreement. Additionally, Koo and Li’s recommendation to interpret the ICC values was followed [26], where ICC < 0.5 indicates a poor correlation, 0.5 ≤ ICC < 0.75 indicates a moderate correlation, 0.75 ≤ ICC < 0.9 indicates a good correlation, and ICC ≥ 0.9 indicates an excellent correlation. The independent samples t test and Chi-square test were performed to compare continuous and categorical variables between the two groups. IBM SPSS Statistics for Windows version 27 (Armonk, NY, USA) was used, and statistical significance was defined as P values less than 0.05.

## Results

The SLE and control group aged 40.33 ± 8.98 years and 38.88 ± 11.01 years, respectively. The majority of participants were female in both groups. Table 1 presents baseline characteristics, including age, sex, body mass index, heart rate, past medical history, habit history, and familial history, indicating no significant differences between the SLE and control groups, showing a proper matching between SLE and control group (all *P* values > 0.05).

**Table 1:** Baseline characteristics of study participants

Characteristics	SLE (*n* = 52)	Control (*n* = 53)	*P* value
Age (years)	40.3 ± 8.9	38.8 ± 11.0	0.46
*Sex*			
Female	50 (96.2%)	45 (84.9%)	0.09
Male	2 (3.8%)	8 (15.1%)	
BMI (Kg/m^2^)	27.2 ± 4.1	27.0 ± 5.0	0.79
Heart rate (/min)	78.5 ± 12.4	76.0 ± 15.0	0.39
History of thrombotic events	1 (1.9%)	1 (1.8%)	1.00
*Co-morbidities*			
DM	2 (3.8%)	4 (7.5%)	0.69
HTN	7 (13.5%)	10 (18.8%)	0.62
DLP	8 (15.4%)	15 (28.3%)	0.17
Smoking status	1 (1.9%)	5 (9.4%)	0.21
Family history of SLE	6 (11.5%)	1 (1.8%)	0.11
*Systemic involvement*	45 (86.5%)		
Musculoskeletal disorders	45 (86.5%)		
Skin disorders	41 (78.8%)		
Hematological disorders	29 (55.8%)		
Nephrology disorders	15 (28.8%)		
Neuropsychiatric disorders	14 (26.9%)		
Reproductive disorders	9 (17.3%)		
Cardiac disorders	2 (3.8%)		
Pulmonary disorders	1 (1.9%)		
*History of medication*			
Prednisolone	50 (96.2%)		
Hydroxychloroquine	32 (61.5%)		
Immunosuppressives	14 (26.9%)		
MTX	11 (21.1%)		
NSAID	8 (15.4%)		
Discontinue medication	21 (40.4%)		

*SLE* systemic lupus erythematosus, *BMI* body mass index, *DM* diabetes mellitus, *HTN* hypertension, *DLP* dyslipidemia, *MTX* methotrexate, *NSAID* nonsteroid anti-inflammatory drug

### Clinical characteristics of the SLE group

The mean duration of SLE diagnosis was 15.11 ± 9.89 years. The most common systemic involvements were musculoskeletal (86.5%) and dermatologic (78.8%) disorders. Among the SLE patients, pericardial effusion was observed in two cases (3%). Prednisolone was the most frequently prescribed medication (92.2%), while nonsteroidal anti-inflammatory drugs (NSAIDs) were the least frequently used (15.4%) (Table 1).

### Echocardiographic findings

We found no significant difference between the SLE and control groups in terms of LVEDV (*P* value: 0.45), LVESV (*P* value: 0.14), LVEF (*P* value: 0.19), left ventricular stroke volume (LVSV) (*P* value: 0.16), left ventricular cardiac output (LVCO) (*P* value: 0.44), left ventricular end-diastolic mass (LVEDM) (*P* value: 0.26), and left ventricular end-systolic mass (LVESM) (*P* value: 0.55). However, we observed statistically significant lower values for LVGLS in the 3D view (*P* value < 0.001), 2D LVGLS in LAX view (*P* value < 0.001), A4C view (*P* value: 0.009), and A2C view (*P* value < 0.001) among SLE patients in comparison with the control group (Table 2).

**Table 2:** Echocardiographic findings of study subjects

Parameters	SLE (*n* = 52)	Control (*n* = 53)	*P* value
LVEDV (mL)	86.8 ± 20.3	89.9 ± 18.9	0.45
LVESV (mL)	34.8 ± 11.7	33.8 ± 9.1	0.14
LVEF (%)	60.5 ± 7.1	62.4 ± 6.2	0.19
LVSV (mL)	52.4 ± 13.0	56.3 ± 13.2	0.16
LVCO (L/min)	4.0 ± 1.0	4.2 ± 1.2	0.44
LVEDM (g)	139.3 ± 12.1	132.4 ± 28.2	0.26
LVESM (g)	141.2 ± 11.7	139.1 ± 13.6	0.55
*LVGLS (%)*			
3D	− 17.3 ± 3.1	− 19.7 ± 2.1	< 0.001
LAX (2D)	− 18.6 ± 7.8	− 22.4 ± 2.8	0.001
A4C (2D)	− 19.4 ± 3.3	− 21.4 ± 3.7	0.009
A2C (2D)	− 18.3 ± 7.0	− 22.1 ± 2.9	< 0.001
Average (2D)	− 19.5 ± 2.8	− 21.9 ± 2.4	< 0.001

*SLE* systemic lupus erythematosus, *LV* left ventricle, *EDV* end-diastolic volume, *ESV* end-systolic volume, *EF* ejection fraction, *SV* stroke volume, *CO* cardiac output, *EDM* end-diastolic mass, *ESM* end-systolic mass, *GLS* global longitudinal strain, *LAX* apical long-axis, *A4C* apical 4-chamber, *A2C* apical 2-chamber

### Inter-rater and intra-rater reliability of the measurements

Our analysis demonstrated an excellent correlation (all ICCs > 0.9) for LVEDV, LVESV, and LVEF measurements. Furthermore, good inter-rater reliability and intra-rater reliability were observed regarding LVGLS measurements using 3D-STE with ICCs of 0.75 and 0.76, respectively (Table 3).

**Table 3:** Inter-rater and intra-rater reliability of echocardiographic measurements in SLE patients

Parameters	Inter-rater ICC	Intra-rater ICC
LVEDV	0.94	0.98
LVESV	0.99	0.98
LVEF	0.94	0.91
LVGLS (3D)	0.75	0.76

*SLE* systemic lupus erythematous, *ICC* intraclass correlation coefficient, *LVEDV* left ventricular end-diastolic volume, *LVESV* left ventricular end-systolic volume, *LVEF* left ventricular ejection fraction, *LVGLS* left ventricular global longitudinal strain

### Comparing other variables

No significant differences were found in the LVGLS measurements in the 3D view among SLE patients who were taking methotrexate, NSAIDs, or immunosuppressive drugs compared to those who were not taking these medications. Similarly, no differences were found in LVGLS measurements between SLE patients who had discontinued their medication and those who were still taking SLE-related medications. However, we observed a significant difference regarding LVGLS measurements between patients taking prednisolone and those not (*P* value: 0.02) (Table 4). Furthermore, SLE patients with nephrologic complications had significantly lower LVGLS measurements in the 3D view (*P*: 0.03) compared to those without nephrologic complications (Table 5).

**Table 4:** Effects of SLE medications on LVGLS

Variable	LVGLS 3D (%)
*Prednisolone*	
Yes (*n* = 50)	− 17.1 ± 2.9
No (*n* = 2)	− 22.0 ± 5.6
*P* value	0.02
*Hydroxychloroquine*	
Yes (*n* = 32)	− 16.8 ± 2.2
No (*n* = 20)	− 17.3 ± 2.7
*P* value	0.30
*Immunosuppressives*	
Yes (*n* = 14)	− 16.5 ± 2.6
No (*n* = 38)	− 17.5 ± 4.2
*P* value	0.41
*MTX*	
Yes (*n* = 11)	− 17.7 ± 2.1
No (*n* = 41)	− 17.1 ± 3.3
*P* value	0.56
*NSAID*	
Yes (*n* = 8)	− 17.7 ± 2.0
No (*n* = 44)	− 17.2 ± 3.2
*P* value	0.72
*Discontinue medication*	
Yes (*n* = 21)	− 17.5 ± 4.0
No (*n* = 31)	− 17.2 ± 2.2
*P* value	0.76

*SLE* systemic lupus erythematosus, *LVGLS* left ventricular global longitudinal strain, *MTX* methotrexate, *NSAID* nonsteroid inflammatory drug

**Table 5:** Effects of SLE systemic involvements on LVGLS

Variable	LVGLS 3D (%)
*Hematological disorders*	
Yes (*n* = 29)	− 16.8 ± 2.3
No (*n* = 23)	− 17.9 ± 3.8
*P* value	0.24
*Pulmonary disorders*	
Yes (*n* = 1)	− 15.0 ± 0.0
No (*n* = 51)	− 17.3 ± 3.1
*P* value	0.45
*Nephrologic disorders*	
Yes (*n* = 15)	− 15.6 ± 4.0
No (*n* = 37)	− 18.1 ± 2.4
*P* value	0.03
*Musculoskeletal disorders*	
Yes (*n* = 45)	− 17.2 ± 3.2
No (*n* = 7)	− 18.1 ± 2.2
*P* value	0.49
*Neuropsychiatric disorders*	
Yes (*n* = 14)	− 15.8 ± 3.9
No (*n* = 38)	− 17.7 ± 2.2
*P* value	0.06
*Reproductive disorders*	
Yes (*n* = 9)	− 17.6 ± 2.1
No (*n* = 43)	− 17.2 ± 3.3
*P* value	0.77
*Skin disorders*	
Yes (*n* = 41)	− 17.2 ± 3.4
No (*n* = 11)	− 17.2 ± 1.8
*P* value	0.95
*Cardiac disorders*	
Yes (*n* = 2)	− 19.0 ± 1.4
No (*n* = 50)	− 17.5 ± 2.7
*P* value	0.44

*SLE* systemic lupus erythematosus, *LVGLS* left ventricular global longitudinal strain

**Fig. 1:**
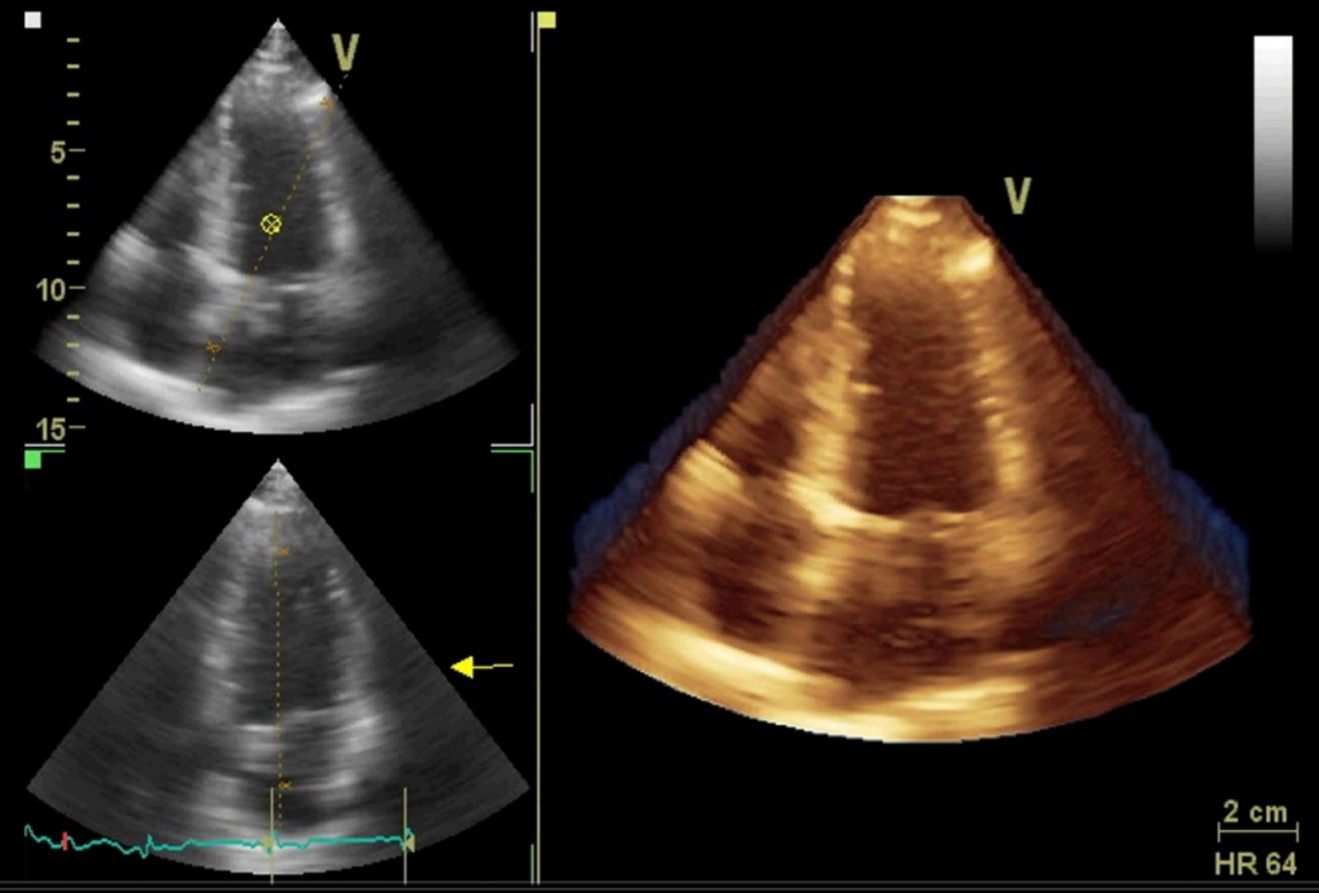
Three-dimensional full-volume echocardiography

Moreover, a positive and significant correlation was found between the duration of SLE disease diagnosis and LVGLS measurements in the 3D view (*r* (50): 0.46, *P* value < 0.001) (Fig. 1).

## Discussion

**Fig. 2:**
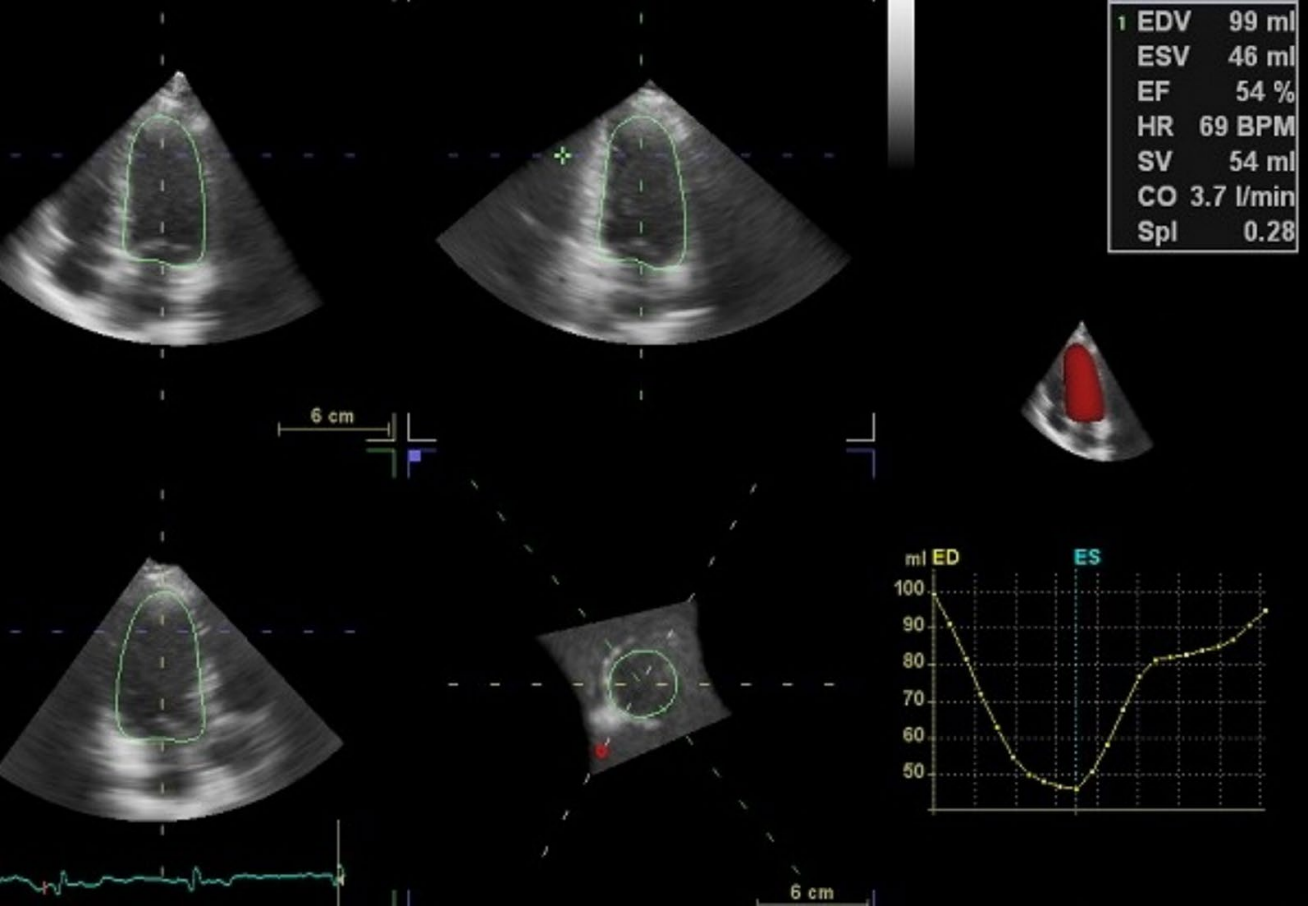
Tracing of the endocardial border is performed, both in the long and short axis of the ventricle in systole and diastole, for volumetric assessment of left ventricle. In right panel, volume time-plot and quantitative analysis and 3D model are presented

**Fig. 3:**
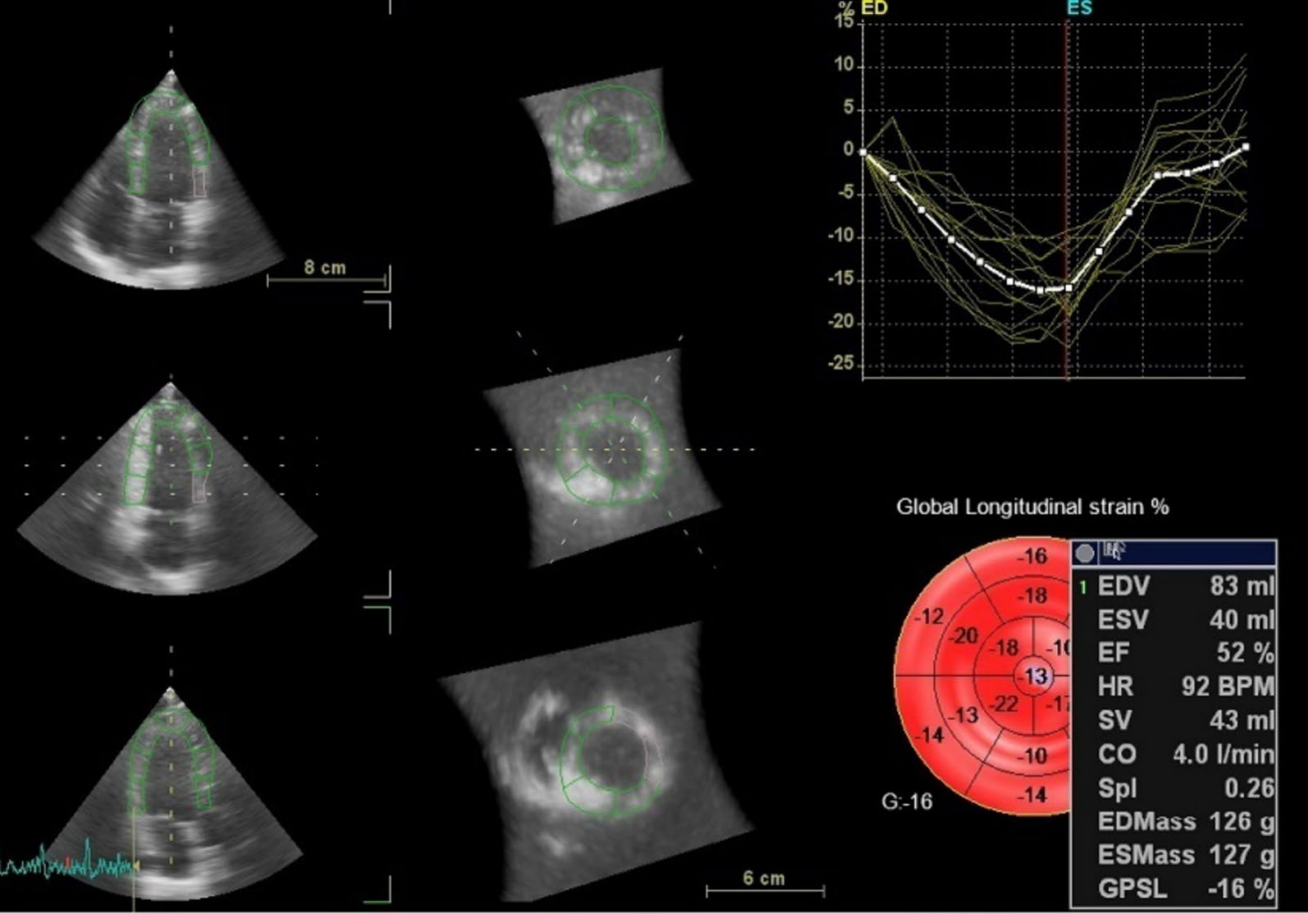
AutoLVQ plane after segmentation process in left panel. Bull’s-eye reconstruction of 3D-LVGLS in right panel

**Fig. 4:**
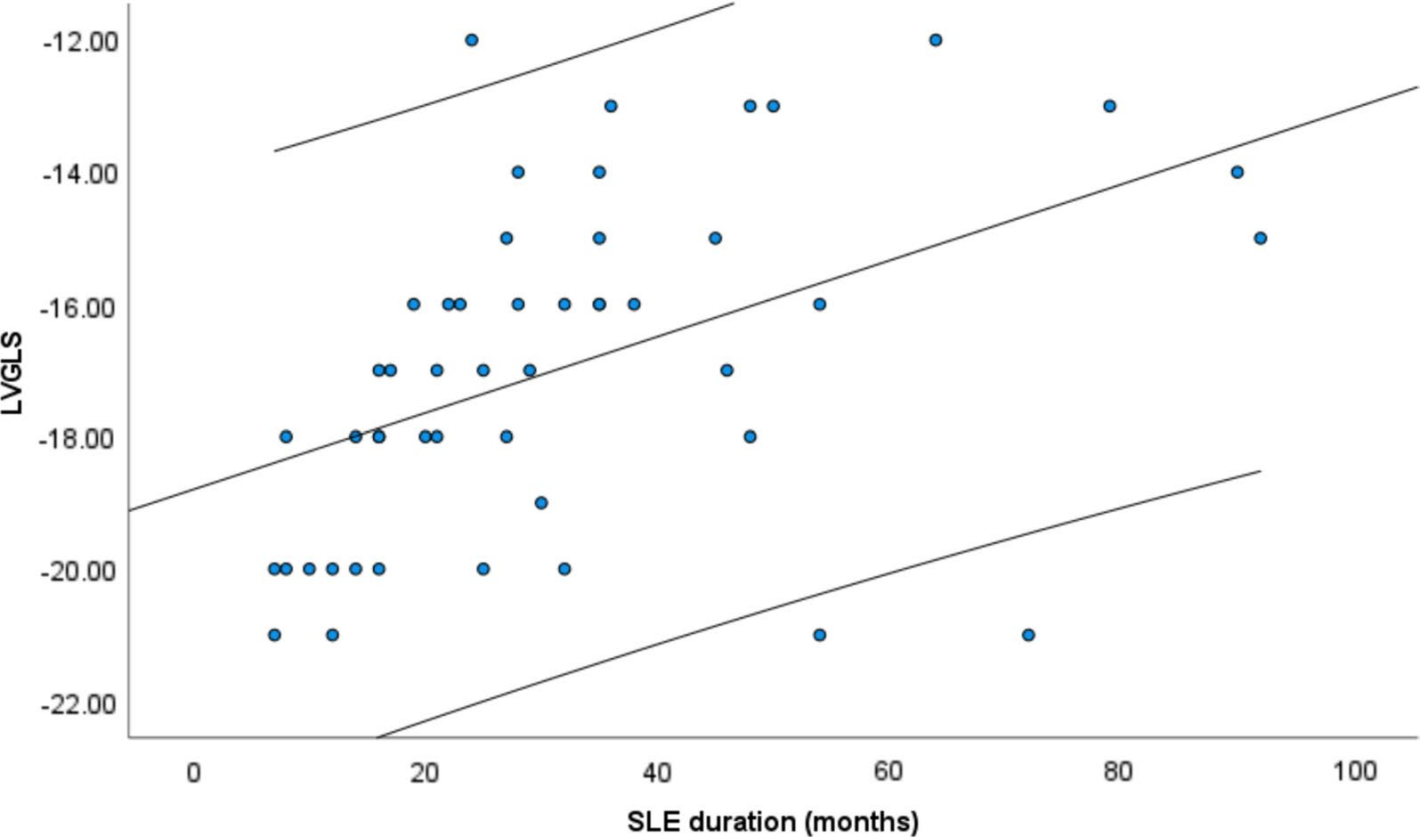
Correlation between LVGLS and SLE duration among study participants

The heightened risk of cardiovascular disease and adverse outcomes in patients with SLE is well established [4, 6, 7]. However, the lack of quantifiable measures of early myocardial damage has hindered the ability to guide interventions [8]. The measurement of LVGLS in a 3D view is a relatively novel, accurate, and operator-independent approach to evaluate LV function with 3D-STE. Despite the potential benefits of LVGLS, there is limited information on its use in SLE patients (Figs. 2, 3, 4).

The current study did not find a significant difference between the SLE patients and controls regarding LVEDV, LVESV, LVEDM, LDESM, and LVEF. Similarly, in a study of 45 SLE patients by Poorzand et al., no considerable difference was found between SLE and control groups concerning LVEF, LVEDV, and LVESV [27]. Nikdoust et al. [28] also showed that LVEF is not markedly affected in SLE patients compared with a healthy population. However, other studies have yielded conflicting results. For example, Deng et al. [13] observed marked increases in LVESV and left ventricular mass (LVM) and a decrease in LVEF in the SLE group compared with the control group, while LVEDV did not differ between the groups. In another study of juvenile-SLE patients, LVEF measurements were not reduced, while LVM was increased compared to healthy individuals [29]. Given the contradictory findings, further extensive studies are needed to draw a definitive conclusion.

LVGLS has been proposed as a sensitive factor for predicting cardiovascular events, such as MI, ventricular hypertrophy, and drug-induced cardiac toxicity [30, 31]. Consequently, LVGLS has gained increased attention as a predictive factor for CVDs in SLE patients. Huang et al. conducted a study in 2014 comparing the LV function of 50 SLE patients and 50 healthy individuals using 3D-STE. They found significantly lower LVGLS measurements in SLE patients [14]. Similarly, Gegenava et al. demonstrated that left ventricular LVGLS was significantly impaired as a marker of systolic impairment in SLE patients and could be utilized as a new tool to predict CVDs in this population [32]. Poorzand et al. and Bulut et al. also reported significantly lower LVGLS measurements in SLE patients compared to healthy controls [27, 33]. Consistent with these findings, our study also revealed markedly reduced LVGLS in the SLE group compared to the control group. While LVEF did not differ between SLE patients and controls, the differences in LVGLS findings suggest that 3D-STE measurement of LVGLS may be a better predictor of CVDs in SLE patients.

The reproducibility of novel methods is often a concern for clinicians. In previous studies, 3D-STE has been shown to be a reliable and precise method for measuring cardiac function in both adult and pediatric populations [34–39]. In agreement with previous researches, our investigation demonstrated favorable inter- and intra-rater reliability levels in evaluating cardiac parameters with 3D-STE. These findings suggest that this imaging technique is a dependable and consistent tool for monitoring and assessing cardiac function in patients over time.

Our study revealed a noteworthy finding regarding the impact of corticosteroid treatment on LVGLS measurements, with patients taking oral corticosteroids showing a significant reduction in LVGLS compared to those not receiving corticosteroids. Previous studies have demonstrated a strong association between corticosteroid use and an elevated risk of adverse cardiovascular events, including MI and angina [6]. Moreover, it has been suggested that corticosteroid use is linked to an increased risk of carotid plaque formation [40, 41], worsened lipid profile, and elevated Framingham score [42–44]. However, limited research has explored the effects of corticosteroids on LVGLS. Aksakal et al. [45] suggested that high-dose intravenous steroid administration may decrease LVGLS.

To the best of our knowledge, there are few studies investigating the impact of SLE-related renal involvement on LVGLS measurements. Our study is also the first to explore this association in an Iranian population. Renal impairment has long been recognized as an underlying cause for traditional cardiovascular risk factors in SLE patients, such as hypertension and dyslipidemia [46, 47]. Moreover, several studies have identified renal dysfunction as an independent nontraditional cardiovascular risk factor [48, 49]. Left ventricular hypertrophy (LVH) is commonly observed in patients with end-stage renal disease, and previous research has indicated a correlation between LVH and reduced LVGLS [50, 51]. Similarly, Krishnasamy et al. [52] reported a significant reduction in LVGLS measurements among patients with renal dysfunction. Lou et al. [53] also found lower LVGLS values among SLE patients with nephrologic impairment. In line with these findings, our study demonstrated a marked decrease in LVGLS among SLE patients with renal involvement.

We also explored the relationship between SLE duration and LVGLS measurements, which, to our knowledge, has not been previously investigated in an Iranian population. We observed a positive correlation between disease duration and LVGLS parameters, consistent with Farag et al.’s research on a group of SLE patients [54]. However, Deng et al. did not find a similar association in their study of 43 SLE patients, which may be attributed to differences in the characteristics of the study population. The exclusion of participants with cardiac, renal, and thyroid dysfunction, and older male and female participants in Deng et al.’s [13] study, may have contributed to the discrepancy in results.

It is important to address some limitations regarding this study. Current study was conducted on a relatively small population; therefore, the results may not be attributable to the broader population of SLE patients. Additionally, due to technical difficulties, we were unable obtain CMR images from participants and compare them with the results of 3D-STE. Furthermore, the cross-sectional design of our study limits the ability to establish causality between SLE and the observed changes in cardiac function. Longitudinal studies are necessary to track changes in cardiac function over time and to evaluate the effects of disease progression and treatment on cardiac function in SLE patients.

## Conclusions

Our study showed that SLE patients had significantly lower LVGLS measurements despite having normal LVEF values compared to healthy individuals. This finding highlights the importance of using more sensitive and accurate tools, such as 3D-STE, in assessing cardiac function in SLE patients. The ability of this technique to detect subtle changes in cardiac function may be especially valuable in predicting future cardiovascular events in this population. Therefore, it is suggested that 3D-STE be considered a valuable adjunct to routine cardiac evaluation in SLE patients.

## Data Availability

The data supporting current study is available from the corresponding author upon reasonable request.

## Abbreviations

SLE: Systemic lupus erythematosus
CVD: Cardiovascular disease
MI: Myocardial infarction
LV: Left ventricle
LVEF: Left ventricular ejection fraction
CMR: Cardiac magnetic resonance imaging
TDI: Tissue Doppler imaging
2D: Two-dimensional
STE: Speckle tracking echocardiography
3D: Three-dimensional
A4C: Apical 4-chamber
LVEDV: Left ventricular end-diastolic volume
LVESV: Left ventricular end-systolic volume
LVGLS: Left ventricle global longitudinal strain
A2C: Apical 2-chamber
LAX: Long-axis
ICC: Intraclass correlation coefficient
NSAID: Nonsteroid anti-inflammatory drugs
LVSV: Left ventricular stroke volume
LVCO: Left ventricular cardiac output
LVEDM: Left ventricular end-diastolic mass
LVESM: Left ventricular end-systolic mass
LVM: Left ventricular mass
LVH: Left ventricular hypertrophy
